# Monte Carlo-Based Radiobiological Investigation of the Most Optimal Ion Beam Forming SOBP for Particle Therapy

**DOI:** 10.3390/jpm13010023

**Published:** 2022-12-22

**Authors:** Ioannis Kantemiris, Eleftherios P. Pappas, Georgia Lymperopoulou, Dimitrios Thanasas, Pantelis Karaiskos

**Affiliations:** 1 Medical Physics Department, Metropolitan Hospital, 18547 Neo Faliro, Greece; 2 Medical Physics Laboratory, Medical School, National and Kapodistrian University of Athens, 11527 Athens, Greece; 3 1st Department of Radiology, Medical School, National and Kapodistrian University of Athens, 11528 Athens, Greece

**Keywords:** particle therapy, ion beams, relative biological effectiveness, heavy ions, Monte Carlo

## Abstract

Proton (p) and carbon (C) ion beams are in clinical use for cancer treatment, although other particles such as He, Be, and B ions have more recently gained attention. Identification of the most optimal ion beam for radiotherapy is a challenging task involving, among others, radiobiological characterization of a beam, which is depth-, energy-, and cell type- dependent. This study uses the FLUKA and MCDS Monte Carlo codes in order to estimate the relative biological effectiveness (RBE) for several ions of potential clinical interest such as p, ^4^He, ^7^Li, ^10^Be, ^10^B, and ^12^C forming a spread-out Bragg peak (SOBP). More specifically, an energy spectrum of the projectiles corresponding to a 5-cm SOBP at a depth of 8 cm was used. All secondary particles produced by the projectiles were considered and RBE was determined based on radiation-induced Double Strand Breaks (DSBs), as calculated by MCDS. In an attempt to identify the most optimal ion beam, using the latter data, biological optimization was performed and the obtained depth–dose distributions were inter-compared. The results showed that ^12^C ions are more effective inside the SOBP region, which comes at the expense of higher dose values at the tail (i.e., after the SOBP). In contrast, p beams exhibit a higher $D_{SOPB}/D_{Entrance}$ ratio, if physical doses are considered. By performing a biological optimization in order to obtain a homogeneous biological dose (i.e., dose × RBE) in the SOBP, the corresponding advantages of p and ^12^C ions are moderated. ^7^Li ions conveniently combine a considerably lower dose tail and a $D_{SOPB}/D_{Entrance}$ ratio similar to ^12^C. This work contributes towards identification of the most optimal ion beam for cancer therapy. The overall results of this work suggest that ^7^Li ions are of potential interest, although more studies are needed to demonstrate the relevant advantages. Future work will focus on studying more complex beam configurations.

## 1. Introduction

The goal of radiotherapy is to increase the dose delivered to the target while sparing the surrounding normal tissues/organs. During the last decades, technological advancements in medical linear accelerators (Linacs) have enabled the clinical implementation of contemporary treatment planning and delivery techniques in photon-based treatments such as Intensity Modulated Radiotherapy (IMRT) [1] and Volumetric Modulated Arc Therapy (VMAT) [2]. These techniques are guided by on-couch imaging systems, such as cone-beam CT (CBCT) or lately by magnetic resonance imaging (MRI) [3]. 

However, the capabilities of Linacs seem to have reached their limits in terms of dose modulation and the future of radiotherapy might be particle therapy [4], even though the latter has been present for a few decades now [5]. The main advantages of ion beams are the dose sparing before and after the target by exploiting the form of the Bragg peak (or the Spread-Out Bragg peak, SOBP) and the delivery of lower integral doses compared to photon beams [6,7,8]. Moreover, ion beams are more effective in killing cancer cells due to the enhanced relative biological effectiveness (RBE) [9].

In general, the physical and biological characteristics of an ion beam are owed to the particle’s mass number resulting in a different clinical output for every ion. Beams commonly used in clinical practice are protons (p) and Carbon ions (C) [10], while the characteristics of Helium (He), Lithium (Li), Beryllium (Be), Barium (B), and Nitrogen (N) ions have also been investigated and found to be of clinical interest [10,11,12,13,14,15,16]. However, it has not been proven whether any of the above is the optimal ion beam, if there is one, for cancer treatment [17]. Towards this purpose, Kramer et al. implemented the ^4^He ion beam in the TRip98 treatment planning system in order to create realistic treatment plans and study any potential advantages [15]. Furthermore, Grun et al. also used TRiP98 coupled with the Local Effect Model (LEM IV [18]) and studied p, ^4^He, and ^12^C ions [14]. The authors concluded that the optimal ion beam cannot exist for each beam configuration and tissue type [14]. In a computational study, depth-dose distributions were calculated and compared for a variety of ion beams (up to a mass number of A = 16) [13]. The authors identified ^8^Be and ^10^B as equally effective to ^12^C for targets lying in small depths. Still, efforts to determine the most optimal beam were inconclusive [13].

From a radiobiological point of view, the problem is associated with accurate RBE predictions in clinical beams, which, however, depend on the Linear Energy Transfer (LET) for all secondary particles, also varying with depth and beam configuration. As an example, LET in ^12^C beams is generally higher compared to p, but ^12^C beams demonstrate relatively higher LET outside the Bragg peak and, therefore, cannot be considered directly superior to p [19]. The main advantage of ^12^C over p is the narrower penumbra, which is counterbalanced by the long fragmentation dose tail after the SOBP [20]. The latter effect might be significant especially for treatments employing two or more beams [19,20].

Monte Carlo (MC) simulations have been proven as an essential tool for calculating LET-related quantities in clinical ion beams [12,13,21,22,23,24]. Physical and radiological characteristics can be studied and compared for ions of any mass number, beam quality, and configuration, for all clinically relevant depths. The FLUKA MC package has been repeatedly employed in such studies [13,23,24,25,26]. In addition, using the Monte Carlo Damage Simulation code (MCDS), the number of Double Strand Breaks (DSBs) induced by each charged particle (primary or secondary) can be calculated by taking into account the particle’s energy and LET [27,28,29]. Thus, combining FLUKA with MCDS, one can derive RBE results for all depths for a given primary ion beam.

In our previous study, FLUKA-based dose and dose-averaged LET distributions were determined for ion beams forming SOBP with primary particles of atomic numbers of 1 up to 8 [13]. In this work, similar calculations are first performed, using a newer version of the FLUKA code (which demonstrates improved accuracy in particle therapy), along with the FLAIR graphical user interface (GUI) [30,31,32]. Furthermore, by employing the MCDS code, RBE distributions are derived based on the LET calculations. However, the main goal of this work is to investigate the most optimal ion beam from a biological perspective. Towards this direction, a biological optimization is performed for each primary ion beam in order to achieve homogeneous biological dose (i.e., dose × RBE) in the entire SOBP area. Determined distributions are presented and compared for a single field SOBP of (5 ± 0.3) cm lying 8 cm deep.

## 2. Materials and Methods

### 2.1. Beams and Geometry

**Figure 1:**
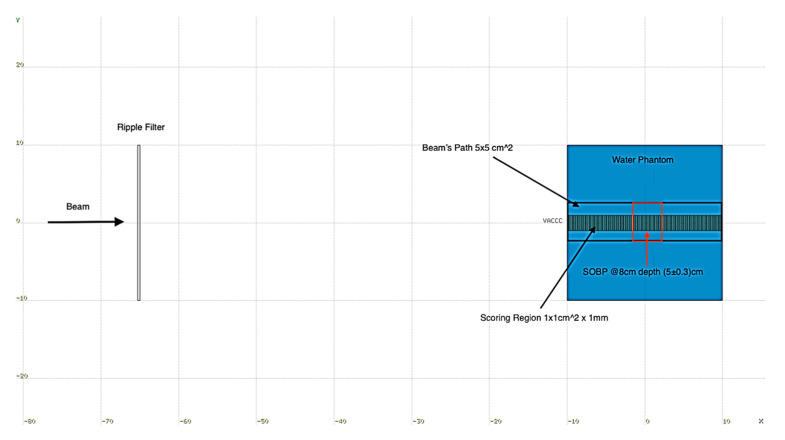
Geometry of the simulations performed throughout this study. The SOBP region is depicted in red. Vacuum (white) is assumed outside the water phantom (blue). Axes are in units of cm.

The isotopes of p, ^4^He, ^7^Li, ^10^Be, ^10^B, and ^12^C were selected to serve as primary ion beams on the grounds of stability (t_1/2_ > 10^6^y) and high abundance. The FLUKA 4-1.0 MC code [25,26,33] was used throughout this study to calculate depth-dose distributions and energy fluence of primary ions and their secondary particles. Simulations involved 5 × 5 cm^2^ fields incident perpendicularly on a 20 × 20 × 20 cm^3^ water phantom. More specifically, a single field SOBP of (5 ± 0.3) cm lying at a depth of 8 cm was configured for each primary ion beam. The SOBP is formed by combining mono-energetic beams of primary ions. More specifically, this is achieved by using pristine peaks combined with a ripple filter [34], as described in Bassler et al. [35]. Vacuum is assumed outside the phantom. The geometry of the simulations is shown with details in Figure 1.

### 2.2. Dose-Averaged LET Calculations

In accordance with our previous study [13], for each ion beam forming a SOBP, the dose-averaged LET was calculated as a function of depth using the following formulas:

(1) $${\bar{LET}}_{D,inf}=\frac{\sum_{i=1}^{Z_{proj}}\sum_{j=1}^{E_{bin}}D\left[E_{j,}Z_{i}\right]\times \frac{dE}{dx}\left[E_{j,},Z_{i}\right]}{\sum_{i=1}^{Z_{proj}}\sum_{j=1}^{E_{bin}}D\left[E_{j,}Z_{i}\right]}$$

where,

(2) $$D\left[E_{j,}Z_{i}\right]=\Phi \left[E_{j,},Z_{i}\right]\times \frac{dE}{dx}\left[E_{j,},Z_{i}\right]$$

while $\Phi \left[E_{j,},Z_{i}\right]$ is the energy fluence and $\frac{dE}{dx}\left[E_{j,},Z_{i}\right]$ is the stopping power of the $Z_{i}$ particle (primary or secondary) having $E_{j}$ energy. All necessary values were adopted from the PSTAR, ASTAR, and MSTAR databases, developed and published by the National Institute of Standards and Technology (NIST) [36].

### 2.3. RBE Calculations

The MCDS code was employed for RBE calculations as a function of depth. This code provides a fast quasi-phenomenological method to interpolate damage yields in DNA from time consuming but detailed track-structures simulations. More specifically, MCDS can calculate the induced DNA damages by the irradiation and presents the results in clusters per gigabase pair of DNA (Gbp) per Gy for all the ions with atomic numbers of Z = 1 up to Z = 54. MCDS can handle energies of a few keV/n up to hundreds of MeV/n. More specifically, the expected number of individual lesions is spatially distributed across a DNA molecule and in the sequel the lesions are grouped into three categories: single strand break (SSB), double strand break (DSB), and base damaged clusters. More details can be found in Ref [37].

In this study, the DSBs of DNA, which is a significant process affecting the cell’s living cycle [38], were scored and used as an indexer of estimating the DSB-based RBE ($RBE_{DSB}$) of ions beams. This approach was adopted from Stewart et al. [37]. Regarding the cell’s oxygenation levels, since the %O_2_ concentration on DSB induction is negligible for ~10% and above [28], the general case of well-oxygenated cells was considered for these calculations. Simulations were performed for a physical dose of 1 Gy.

The total biological effectiveness of a beam is due to the corresponding effectiveness of the secondary particles and nuclei that are produced. In order to calculate the $RBE_{DSB}$ of p, ^4^He, ^7^Li, ^10^Be, ^10^B, and ^12^C, their biological effectiveness is compared to ^60^Co photon beam. Specifically, for each beam and for all depths of clinical interest, d, the $RBE_{i}^{DSB}$ of all the secondary particles, was calculated by:

(3) RBEiDSBd=YieldidYieldC60o=∑j=1EbinDiEj×DSBiEj∑j=1EbinDiEjYieldC60o

where,

(4) $$D\left[E_{j}\right]=\Phi \left[E_{j}\right]\times \frac{dE}{dx}\left[E_{j,}\right]$$

and YieldC60o=8.28584 as calculated by MCDS taking into account the secondary energy spectrum of electrons derived when ^60^Co photons incident on single layer cells. The methodology has been described in Hsiao et al. [39].

The (total) $RBE_{DSB}$ for a primary ion beam as a function of depth, d, can be then calculated by:

(5) $$RBE_{DSB}\left(d\right)=\frac{\sum_{i=1}^{Z_{proj}}D\left(Z_{i}\right)\times RBE_{i}^{DSB}\left(Z_{i}\right)}{\sum_{i=1}^{Z_{proj}}D\left(Z_{i}\right)}$$

According to the above formula for $RBE_{DSB}\left(d\right)$ determination, at a given depth, the contribution of each secondary particle to the total $RBE_{DSB}$ is weighted according to the particle’s local dose contribution, as in Refs [29,37].

### 2.4. Biologically Optimized SOBP

The final step of this work is to present the biologically optimized SOBP dose distributions after taking into account the extracted $RBE_{DSB}$ as a function of depth. The optimization process involved the re-calculation of the relative intensities of the primary mono-energetic beams contributing to the SOBP dose plateau, in order to achieve a homogeneous biological dose in the SOBP, after taking into account the local $RBE_{DSB}$ values. In other words:

(6) $$D_{bio}\left(d\right)=D_{phys}\left(d\right)\times RBE_{i}^{DSB}\left(d\right)$$

with

(7) $$D_{phys}\left(d\right)=\sum_{i=E_{init}}^{E_{final}}f_{i}\times D_{i}\left(d\right)$$

where $f_{i}$ is the relative intensity of the corresponding mono-energetic beam delivering $D_{i}\;$ dose as function of depth, d, for a specific ion beam.

### 2.5. Simulation and Scoring Parameters

A custom-made routine was implemented to calculate the weight of each ion energy in order to achieve a homogeneous (95–107%) dose within the SOBP [40]. The HADROTHErapy default settings were applied, which include a particle transport threshold of 100 keV, with the exception of low energy neutrons with energy cut-off transportation 10^−5^ eV, ensuring increased calculation accuracy [31]. The activated FLUKA physics models enable continued energy loss, energy struggling, δ-ray production and transportation, multiple Coulomb scattering, leptons, photons, hadrons, and ions nuclear interactions (PEANUT model [41], RQMD-2.4 model [42], and BME model [43]).

The track length fluence estimate of each individual particle was scored with a spatial resolution of 1 mm, integrated over a 1 × 1 cm^2^ area, utilizing USRTRACK in combination with custom FLUKA routines. The overall estimated statistical error is <5% (mainly emanating from FLUKA calculations) in both the plateau and SOBP regions. Regarding the dose tail (i.e., after the SOBP), the calculated statistical uncertainties were up to 10% for all the beams considered, except for ^4^He particles. Regarding the latter, due to the negligible dose after the SOBP, uncertainty exceeds 10% after the first 2 mm of dose tail. Therefore, corresponding results are not presented in this study.

Compared to our previous work [13], calculations were carried out using a new version of FLUKA (v.4-1.0) in which the accuracy of the dose distributions of various ion species is significantly refined [26]. This is due to the implementation of Boltzmann Master Equation theory (BME model) [44]. Its main advantage is that it more accurately handles ions with energy below 150 MeV/n.

All simulations were performed by a local workstation equipped with 32 computational threads, clocked at 2.4 GHz. Depending on the mass number of the primary ion beam investigated, the number of starting particles varied from 3 × 10^6^ up to 2 × 10^7^.

## 3. Results

**Figure 2:**
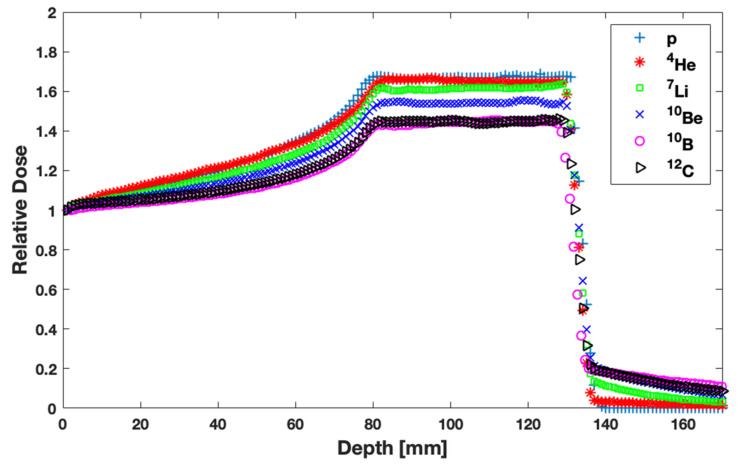
Percentage depth-dose curves (physical dose) for all single field ion beams considered in this study. Data have been normalized at the entrance dose.

Physical doses delivered by p, ^4^He, ^7^Li, ^10^Be, ^10^B, and ^12^C ion beams forming SOBP are illustrated in Figure 2 as a function of depth. Results are normalized at the entrance of the beam. Lighter ions present better physical dose deposition along the beam axis, i.e., higher $D_{SOPB}/D_{Entrance}$ and a smaller or negligible dose tail. If a physical dose distribution is only investigated, p and ^4^He beams seem to be superior to all others considered.

**Figure 3:**
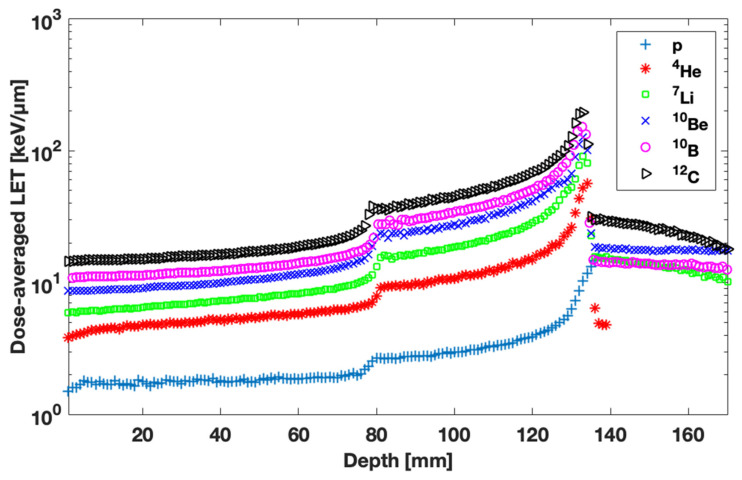
Dose-averaged LET as a function of depth for all ion beams considered in this study.

Using the scored energy fluence, $\Phi \left[E_{j,},Z_{i}\right]$, combined with the associated stopping power ratios, $\frac{dE}{dx}\left[E_{j,},Z_{i}\right]$, dose-averaged LET results were calculated and are presented in Figure 3.

**Figure 4:**
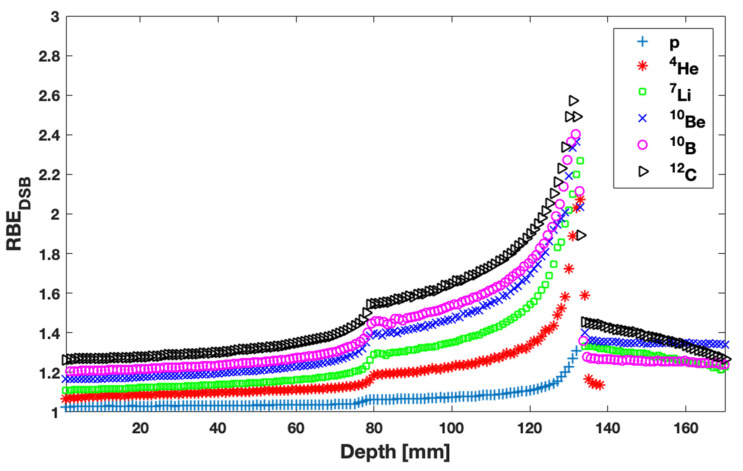
$RBE_{DSB}$ values of p, ^4^He, ^7^Li, ^10^Be, ^10^B, and ^12^C as a function of depth for a well-oxygenated target.

By combining FLUKA results with calculations by the MCDS code and assuming a well-oxygenated target, (total) $RBE_{DSB}$ values as a function of depth are presented in Figure 4. For all ion beams considered, $RBE_{DSB}$ greatly increases at the end of the SOBP area, indicatively exceeding 2.5 for the ^12^C ions.

As a validation step, $RBE_{DSB}$ calculations, presented in Figure 4, were compared with published RBE values wherever available. Regarding p beams, Paganetti reported RBE for a range of LET [45]. Indicatively, for p beam LET in the range of 3–6 keV, this work concluded that $RBE_{DSB}$ = 1.13 ± 0.06, while Paganetti reported 1.1 ± 0.03 [45]. Similarly, for LET in the ranges of 2–3 keV and 6–9 keV, the $RBE_{DSB}$ values determined in this study were 1.07 ± 0.05 and 1.26 ± 0.06, respectively. These results are consistent and within uncertainties with the corresponding published values (1.12 ± 0.05 and 1.35 ± 0.1, respectively [45]). However, for LET in the range of 9–15 keV (i.e., the last 2 mm), $RBE_{DSB}$ values determined herein are considerably lower as compared to Paganetti (i.e., 1.31 ± 0.13 compared to 1.7 ± 0.18 [45], respectively). This may be attributed to volume averaging effects in our simulations and the increased statistical uncertainties within the specific scoring voxel. Regarding the heavier than p ion beams, a number of papers reporting on RBE values have been identified. More specifically, $RBE_{DSB}$ values for ^4^He, ^7^Li, and ^12^C were compared with the corresponding ones published in Refs [12,15,20], respectively. Checking for RBE at characteristic depths of the PDD curves, values reported herein are in excellent agreement with the corresponding published ones, including both low and high LET regions. This comparison served as a validation step for the $RBE_{DSB}$ values used for the subsequent biological optimization.

**Figure 5:**
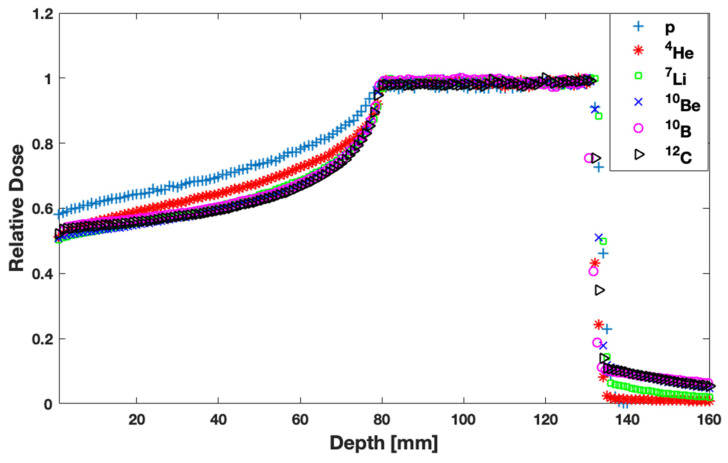
Biologically optimized percentage depth-dose curves for all ion beams considered in this study.

A biological optimization was performed in order to obtain a homogeneous biological dose in the SOBP (i.e., after taking into account the local $RBE_{DSB}$ values shown in Figure 3). Results are presented in Figure 5.

## 4. Discussion

In this simulation study, dose-averaged LET was calculated as a function of depth for ion beams up to an atomic number of 6 (Figure 3). In our previous publication, similar calculations were presented [13]. From a methodological point of view, the main difference of this work compared to the previous one is that a new version of FLUKA was used, which more accurately handles particles with energy below 150 MeV/n. The dose-averaged LET results presented in Figure 3 are consistent with the ones published in our previous study [13], although notable deviations can be detected at the dose tail. Thus, the refinement of our results, due to the usage of the newer version of FLUKA, is confined only after the SOBP region. Furthermore, more recent publications increase the reliability of our calculations for p and alpha particle beams [15,45].

With respect to p beams, according to a literature review paper [45], the average RBE in the entrance region of the SOBP is ~1.1, which rises up to ~1.15 in the center and 1.35 at the distal edge. The $RBE_{DSB}$ index calculated in this study is in good agreement with these results within our statistical uncertainties. A more detailed comparison was presented in Section 3.

Regarding heavier ion beams, Kramer et al. calculated ^4^He RBE with TRiP98 TPS and validated their results by irradiating Chinese hamster ovary cells (CHO-K1) [15]. Our findings are in good agreement with this work; however, due to the high uncertainty at the dose tail region, no RBE values are presented for 2 mm and beyond the distal part of SOBP. ^7^Li beams have been studied in Burigo et al. [12]. They used a microdosimetric—kinetic model and microdosimetric spectra measured by Tissue Equivalent Proportional Counters (TEPC) at NIRS (Japan) and GSI (Germany) and performed MC calculations using the Geant4 code. A comparison with the $RBE_{DSB}$ results presented herein is only meaningful in the plateau and in the peak region because of the different beam quality, i.e., mono-energetic vs. poly-energetic beams. Despite this limitation, our results are consistent with the published values [12]. Furthermore, in a review paper by Suit et al., RBE data were determined for ^12^C ions along with relatively increased uncertainties [20]. $RBE_{DSB}$ values as a function of depth presented in this work (Figure 4) are in close agreement with the average values in Suit et al. [20], except for the distal part of the SOBP. The latter remark can be attributed to the fact that our methodology underestimates the RBE due to volume averaging effect at the high dose gradient areas. Still, a fair agreement is achieved if uncertainties are taken into account. To the best of our knowledge, there is no available RBE data for ^10^Be and ^10^B ion beams.

RBE is an essential index in order to select an ion beam. As expected, according to Figure 4, the effectiveness of a beam increases with increasing atomic number. Thus, ^12^C is the most biologically effective beam among the ones studied in this work. Another essential factor for ion beam selection is the physical dose deposition (Figure 2). From this perspective, p and ^4^He ions seem to be superior to the other (heavier) ones. However, the final outcome is associated with the combination of both RBE and physical dose. This remark led to the calculation and comparison of the biologically optimized dose distributions, shown in Figure 5. The presented results reveal that the healthy tissue before the target is spared better with heavier ions like ^12^C in a single-field irradiation. Moreover, healthy tissues are spared better when irradiated with heavier ions because of the negligible lateral spread of the dose resulting to smaller field sizes compared to proton beams in order to cover the same target volume [20,35,46]. On the other hand, the dose tail of heavier ions and especially of ^12^C ions remains the highest at the first 1 cm after the distal part of SOBP among all the ions studied in this work. In the case of two or more fields, the cumulative dose burden from the tails will be even more pronounced. Therefore, multi-field irradiations will benefit more the distributions of shorter dose tails, as compared to ^12^C. 

**Figure 6:**
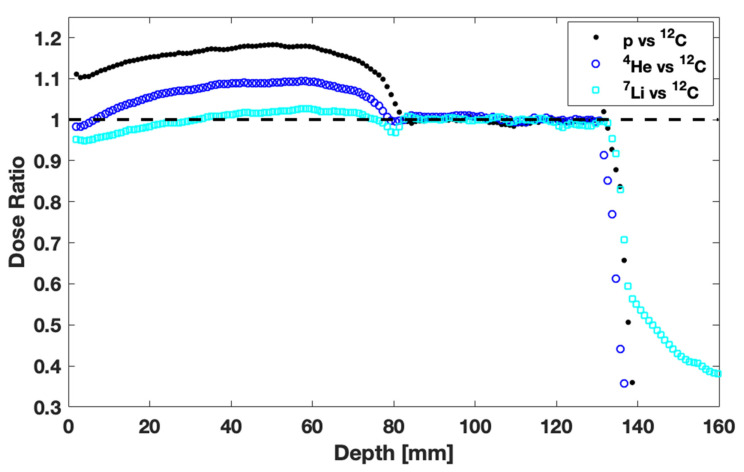
Comparison of p, ^4^He, ^7^Li, and ^12^C ion beams. The ratio of corresponding biologically optimized doses, normalized at the center of the SOBP, is presented as a function of depth.

Apart from the ^12^C and proton beams, which are the two extreme cases of this study and are in clinical use, other particles are also gaining attention from the scientific community [13,14,15,46]. In an effort to reveal potential advantages of such beams, a comparison of the biologically optimized depth-dose curves for p, ^4^He, ^7^Li, ^10^Be, ^10^B, and ^12^C ions was performed. Our results indicate the potential advantage of ^7^Li ions over the other beams and especially over ^12^C. Specifically, ^7^Li ions demonstrate a biological dose distribution similar to ^12^C ions until the distal part of SOBP but a lower dose tail (Figure 5). In order to clearly illustrate this difference, the ratio of the corresponding biologically optimized depth-dose curves (normalized at the center of SOBP) is presented in Figure 6. Before the SOBP, both ion beams (^7^Li and ^12^C) deliver similar biological dose distributions (i.e., a ratio of 1 ± 0.05, which is within statistical uncertainties for most depths evaluated). On the other hand, there is a significant dose reduction at the tail region in favor of ^7^Li (ratio ≪1), as can be seen in Figure 6. In other words, in terms of sparing critical organs, ^7^Li and ^12^C ion beams are comparable in the dose plateau region but ^7^Li is superior in the dose tail. This benefit would be even more pronounced in multi-field beam configurations. For comparison, the corresponding dose ratios of p over ^12^C and ^4^He over ^12^C are also included in Figure 6. There is a clear advantage of both p and ^4^He over ^12^C in terms of healthy tissue sparing at the tail (after the SOBP). However, the benefit is counterbalanced by the considerable over-dosage in the plateau region for both p and ^4^He ions as compared to ^12^C (i.e., ratio > 1 in Figure 6). 

Regarding the methodology employed and the results presented in this work, a number of limitations should be underlined. First of all, this is a purely computational study based on FLUKA and MCDS codes. Thus, determined RBE values do not account for the characteristics of a specific cell type or biological endpoint. Sublethal damage repair kinetics of specific cells have not been considered as well. On the other hand, in a quest to identify the most optimal ion beam for cancer treatment, limiting our results to the characteristics of a specific cell type would correspond to a narrow range of clinical applications. Nevertheless, the presented $RBE_{DSB}$ values are in good agreement with published results wherever available. This work focused only on single-SOBP beams, the basis of all treatment delivery techniques, in order to better illustrate the underlying advantages and drawbacks at a fundamental level. Investigation of biologically optimized dose distributions for more complex irradiation schemes was not performed but will be considered in our future work. Furthermore, technical and technological aspects of delivering ion beams (beyond ^1^p and ^12^C) for clinical use were not considered, nor discussed. Development of such facilities is very challenging, and the advantage or benefit of a beam should be clear enough already from computational studies (such as the present one), to justify the associated costs and research burden for the scientific community. Theoretical and pre-clinical studies should also precede the clinical introduction of a new radiotherapy treatment modality, further increasing the necessary investments.

In this work, a framework was developed for studying ion beams from a radiobiological point of view. The ^7^Li ion beam was identified as a potential candidate for cancer treatment, exhibiting a superior biological dose distribution at the dose tail, as compared to ^12^C. This remark is also in-line with a previous investigation, although mono-energetic beams were studied and no RBE calculations were involved [11]. However, more studies are still needed towards the determination of the most optimal ion beam for clinical use. Future work will focus on quantifying the benefit from using two or more ^7^Li beams or other more complicated beam configurations. If this is proven, incorporating the biological and radiobiological characteristics of specific cell types of clinical interest seems to be the next step in this quest.

## 5. Conclusions

Towards the identification of the most optimal ion beam from a radiobiological point of view, a computational methodology was developed and implemented in order to calculate the $RBE_{DSB}$ index as a function of depth for a variety of ion beams forming SOBP. The obtained results were found consistent with RBE values published in the literature wherever available.

To further characterize the radiobiological characteristics of the beams considered, a biological optimization was performed in order to obtain a homogeneous biological dose (i.e., dose × RBE) in the SOBP area, for all ion beams. Comparison of resulting distributions revealed that the main drawback of ^12^C ions is the increased biological response after the SOBP, i.e., the dose tail. On the other hand, ^12^C ions exhibit a better (biologically optimized) $D_{SOPB}/D_{Entrance}$ ratio compared to all other beams. However, the ^7^Li ions conveniently combine a lower dose tail and a $D_{SOPB}/D_{Entrance}$ ratio similar to ^12^C (Figure 6). In a clinical application employing two or more beams forming SOBP, this advantage of ^7^Li ion beams would result in a more pronounced radiobiological effect.

More studies are needed to clearly identify the most optimal ion beam for clinical applications. Overall results of this work suggest that ^7^Li ions are of potential interest. 

## Data Availability

The data presented in this study are available on request from the corresponding author.
